# Mathematical Problems for Complex Systems

**DOI:** 10.1155/2015/605172

**Published:** 2015-06-24

**Authors:** Haijun Jiang, Haibo He, Jianlong Qiu, Qiankun Song, Jianquan Lu

**Affiliations:** ^1^College of Mathematics and System Sciences, Xinjiang University, Ürümqi, China; ^2^University of Rhode Island, Kingston, RI, USA; ^3^College of Science, Linyi University, Shandong, China; ^4^Department of Mathematics, Chongqing Jiaotong University, Chongqing 400074, China; ^5^Department of Mathematics, Southeast University, Nanjing 210096, China

As most of practical systems have high complexity, complex systems have become a rapidly growing area of mathematics and attracted many researchers. The study of complex systems not only has an important theoretical interest but also is motivated by problems from applied mathematics including physics, chemistry, astronomy, technology, and natural and social sciences. It should be noted that some major problems have not been fully investigated, such as the behavior of stability, synchronization, bifurcation, and chaos control for complex systems, as well as their applications in, for example, communication and bioinformatics.

The special issue contains seven papers; of these, three of the papers are related to application analysis of complex systems to the real world problems. One paper studies the synchronization of chaotic complex systems with fractional-order. One paper investigates the consensus problem for nonlinear complex systems. Another paper provides an approach to determine the unique 3-uniform linear hypertree with the maximum Estrada index. Finally, a paper provides interior principles to calculate the leading elements of the aliased effect-number pattern.

In the paper “Results for Two-Level Designs with General Minimum Lower-Order Confounding,” the authors study the interior principles of calculating the leading elements in _1_^#^ *C*_1_ and _2_^#^ *C*_2_ aliased effect-number pattern. Also, their mathematical formulations are obtained for every lower-order confounding 2^*n*−*m*^ design according to the two different cases.

In the paper “On the Maximum Estrada Index of 3-Uniform Linear Hypertrees,” authors give some basic definitions on the Estrada index of hypergraph and then formulate an algorism for determining the unique 3-uniform linear hypertree with the maximum Estrada index.

In the paper “Consensus of Nonlinear Complex Systems with Edge Betweenness Centrality Measure under Time-Varying Sampled-Data Protocol,” by constructing a suitable Lyapunov-Krasovskii functional and using linear matrix inequality technique, the authors propose a new consensus criterion for nonlinear complex systems with edge betweenness centrality measure. Finally, a numerical example is provided to illustrate the effectiveness of the proposed consensus schemes.

In the paper “One Adaptive Synchronization Approach for Fractional-Order Chaotic System with Fractional-Order 1 < *q* < 2,” based on a new stability result of equilibrium point in nonlinear fractional-order systems, the authors investigate the adaptive synchronization for the fractional-order Lorenz chaotic system with fractional-order 1 < *q* < 2. Numerical simulations show the feasibility of the proposed adaptive synchronization scheme.

In the paper “A New Chaotic Map and Its Application on Image Encryption,” the authors present a novel approach to create the new chaotic map and then applied it to image encryption. Compared with traditional classic one-dimensional chaotic map like Logistic Map and Tent Map, this newly created chaotic map demonstrates many better chaotic properties for encryption. The simulation results and security analysis show that such method not only meets the requirement of imagine encryption but also has better security, which is very useful for general applications.

In the paper “Description and Application of a Mathematical Method for the Analysis of Harmony,” after briefly introducing the basic concepts of harmony theory, the authors expound the five essential elements for the quantitative description of harmony issues in water resources management: harmony participant, harmony objective, harmony regulation, harmony factor, and harmony action. Furthermore, a basic mathematical equation for the harmony degree is introduced.

In the paper “A Learning Framework of Nonparallel Hyperplanes Classifier,” the authors concerned the learning framework of nonparallel hyperplanes support vector machines (SVM) for binary classification and multiclass classification. The given framework not only includes twin SVM and its many deformation versions but also extends them into multiclass classification problem with loss functions or different parameters. The numerical experiments on several artificial and benchmark datasets indicate that the introduced frameworks not only are fast but also have good generalization.

